# The impact of myostatin variants on growth traits in South African Bonsmara beef cattle

**DOI:** 10.1007/s11250-024-04208-3

**Published:** 2024-10-25

**Authors:** Rendani Madula, Carina Visser, Este van Marle-Köster

**Affiliations:** Department of Animal Science, University of Pretoria, Pretoria, South Africa

**Keywords:** Beef cattle, Diagnostic testing, Double muscling, Efficiency, Genotype, Myostatin

## Abstract

Double muscling occurs when the myostatin (*MSTN*) gene is deactivated due to a series of mutations, leading to uncontrolled muscle growth and excessive muscle fiber accumulation, as the gene can no longer effectively regulate muscle development. This study aimed to assess the impact of *MSTN* variants and their combinations on growth traits, namely direct birth weight (BW_DIR_), direct weaning weight (WW_DIR_), average daily gain (ADG) and feed conversion ratio (FCR) in the South African (SA) Bonsmara. Genomically enhanced estimated breeding value (GEBVs) for traits of interest, and *MTSN* genotypes for SA Bonsmara animals were available for the study. Thirteen *MSTN* variants (Nt821, Q204X, F94L, E226X, E291X, C313Y, Nt419, S105C, D182N, Nt414, Nt324, Nt267, and Nt748) were routinely genotyped using the IDBv3 SNP array. Genotypic frequencies of *MSTN* variants ranged from 1.18% for Q204X to 35.02% for Nt748. No association was observed between the Nt267 variant and any growth traits, while both Nt748 and Nt414 variants affected WW_DIR_, ADG and FCR (*p* < 0.05). The results of the effect of multiple variants on growth traits indicated that there was an additive effect when more than one *MSTN* variant was present in an individual. This study is the first study to report the impact of *MSTN* variants on traits of economic importance in the SA Bonsmara breed.

## Introduction

Myostatin, also known as growth and differentiation factor 8 (GDF-8), is a protein produced and secreted by myocytes that inhibits muscle growth in young animals (Elkina et al. 2011; Sharma et al. 2015). The *MSTN* gene plays a crucial role in regulating muscle mass homeostasis (Dominique and Gérard 2006) and adipogenesis (Deng et al. 2017). Notably, various genetic variants have been identified that inactivate the *MSTN* gene, leading to a loss of its ability to inhibit muscle fiber growth. This results in uncontrolled muscle growth, as seen in cattle with double muscling (McPherron and Lee 1997; Grobet et al. 1998).

In 1995, the causative mutation of muscular hypertrophy was mapped to bovine chromosome (BTA) 2 using linkage analysis, providing strong evidence that this locus was the single, autosomal, major gene underpinning the double muscling phenotype (Charlier et al. 1995). Up to 20 mutations have since been identified in the bovine *MSTN* gene (Aiello et al. 2018; Haruna et al. 2020) and variants include deletions, insertions, and nucleotide substitutions that limit the activity of the *MSTN* gene (Aiello et al. 2018). Several genetic variants in *MSTN* coding regions (exons 1 to 3) have been characterized as silent and cause non-synonymous changes (Hunt et al. 2009; Konovalova et al. 2021), while polymorphisms in the promoter region of the *MSTN* gene have been shown to affect meat quality, growth and reproduction traits in several cattle breeds (Fiems 2012; Druet et al. 2014). Currently, there are thirteen known genetic variants that are included in routine DNA testing, namely Nt821 (commonly known as *del11*), C313Y, E226X, Q204X, E291X, Nt419, D182N, S105C, Nt414, Nt324, Nt267, Nt748 and F94L, which are known to restrain and decrease the activity of myostatin protein, causing double muscling (Dunner et al. 2003; Bellinge et al. 2005). Variants such as Nt821 and Q204X are known as detrimental variants (Purfield et al. 2020) resulting in heavier birth weights, slower growth and reduced fertility compared to other variants such as F94L (Csürhés et al. 2023). Nt821 and Q204X have also been classified as disruptive variants in Belgian blue cattle (Druet et al. 2014; Anwar et al. 2020) and Charolais breeds (Keogh et al. 2021; Csürhés et al. 2023). The F94L variant is generally seen as a positive mutation causing intermediate muscling (Csürhés et al. 2023) and greater average daily gain (Hales et al. 2020), as well as increased muscle mass, carcass yield and carcass yield without any associated reproductive problems in Limousin heifers (Lee et al. 2019; Anwar et al. 2020; Csürhés et al. 2023). It is, therefore; important to note that different cattle breeds may carry different *MSTN* mutations, and that their effect may vary between different genotypes.

The general role of the *MSTN* gene in the expression of growth and muscle development is evident from literature, but the specific impact of various variants has not been quantified in SA beef breeds. Despite being discriminated against by some breed societies in South Africa due to its drawbacks such as high birth weights resulting in increased incidences of dystocia, double-muscled cattle are accepted across the world due to certain advantages such as a higher dressing percentage, higher lean meat content and lower fat content (Wiener et al. 2009; Allais et al. 2010; Webb and Casey 2010). The prevalence of specific variants in a breed, and their association with traits of economic importance, may guide farmers with regards to selection decisions. South African Bonsmara cattle are known for their growth performance, achieving average daily gains of 1.95 kg/day (Jiyana 2019), making them well-suited to the country’s extensive grazing systems. This superior growth rate, combined with their hardiness and adaptability, has contributed to their popularity among local cattle producers. The objective of this study was to assess the potential impact of the *MSTN* variants present in SA Bonsmara cattle on growth traits.

## Materials and methods

### Materials

Consent and ethical approval for the use of the data for the study was granted by the SA Bonsmara Breeder Society and the University of Pretoria Ethics committee (NAS223/2020), respectively. A total of 1778 Bonsmara animals that were routinely genotyped for double muscling were genotyped between the years 2018 and 2021 using the International Dairy Beef (IDBv3) SNP array (Mullen et al. 2013) at Weatherbys in Ireland. This array features 53 714 SNP probes distributed across the bovine genome with an average spacing of 37.4 kb, as well as nine *MSTN* SNP variants within the *MSTN* gene located on BTA2. The following thirteen *MSTN* variants were included for diagnostic testing of muscular hypertrophy namely, Nt821, Q204X, F94L, E226X, E291X, C313Y, Nt419, S105C, D182N, Nt414, Nt324, Nt267, and Nt748.

The genotypic results were coded per variant with 0, 1 or 2; with 0 indicating that the animal was homozygous for the normal alleles at the specific locus, 1 indicating a heterozygous carrier of the specific mutation; and 2 indicating homozygous status for the double muscled (affected) mutation at the specific locus.

Genomic estimated breeding values (GEBVs) which are part of routine genetic evaluations for the SA Bonsmara breed, were available for all the animals that were genotyped. The GEBV data set included four traits namely, direct birth weight (BW_DIR_), direct weaning weight (WW_DIR_), average daily gain (ADG) and feed conversion ratio (FCR).

## Analyses

The genotypic data containing the *MSTN* variants and the GEBVs for growth traits for SA Bonsmara breed were provided in Excel format. Animals with missing ID (*n* = 1278), as well as duplicates (*n* = 233), were removed from the raw data. Genotypic frequencies for SA Bonsmara per genetic variant was estimated using Microsoft Office Excel (Microsoft 2016). The Listwise deletion method was used to eliminate missing data records.

Statistical analysis was conducted using Statistical Package for Social Sciences (SPSS) software (version 27; IBM), where descriptive statistical analysis parameters were estimated and reported as mean ± standard error (SE). The SPSS software was configured to a standard confidence level of 95% and independent t-test was used to identify the statistical differences between groups of animals. The normality of the data was considered using the Shapiro–Wilk test with Levene test used to determine equal variances, and log transformations (log10) were performed where normal distribution was violated. Prior to completing the multivariate analysis of variance (MANOVA) for the growth traits, it was necessary to test the assumption that there is no collinearity among the dependent variables (i.e. growth traits). Pearson correlations between all of the dependent variables confirmed the absence of collinearity as all the correlation coefficients were less than 0.80 (ranging between − 0.773 and 0.746) as recommended by Pallant (2020).

Data which included traits and genotypes were statistically analyzed by means of two-way MANOVA using the General Linear Models (GLM) procedure of SPSS version 27 (Pallant 2020). The following equation for GLM model for the effects of individual genetic variants of *MSTN* on growth traits of interest was used for analysis:

$${{\rm{Y}}_{\rm{k}}}{\mkern 1mu} {\rm{ = \alpha + }}{\mkern 1mu} {{\rm{\beta }}_{\rm{1}}}{{\rm{X}}_{\rm{1}}}{\mkern 1mu} {\rm{ + }}{\mkern 1mu} {{\rm{\beta }}_{\rm{2}}}{{\rm{X}}_{\rm{2}}}{\rm{ + }} \ldots {\rm{.}}{\mkern 1mu} {\rm{\beta }}{{\mkern 1mu} _{\rm{k}}}{{\rm{X}}_{\rm{k}}}{\rm{ + E}}$$

Where.

Y (dependent variables) is growth traits (birth weight, weaning weight, average daily gain and feed conversion ratio).

α is the coefficient model of the model.

β is the beta coefficient of the X’s variables in the model.

X (independent variable) is the variants (Nt748, Nt414, Nt267 and Q204X).

E is the error term.

K is the k^th^ trait under growth.

The Bonferroni multiple range test was applied where data was imbalanced to reduce the incidence of false positive results. Nt748, Nt414, Nt267, Q204X variables for Bonsmara animals were modelled as fixed factors and BW_DIR_, WW_DIR_, ADG, FCR variables were modelled as covariates. Differences between means were tested by means of the Least Significant Difference (LSD) multiple range test. The statistical differences were considered significant at a probability level of 5% (*p* < 0.05).

For animals that presented with more than one variant in the *MSTN* gene, combination codes were generated to test for their combined effects on traits of interest in this study. The various combinations of four *MSTN* variants (Nt748, Nt414, Nt267 and Q204X) observed in SA Bonsmara animals were coded as indicated in Table 1.

**Table 1:** The combinations of the genetic variants identified in the genotyped SA Bonsmara animals

	MSTN variant combination^1^				
Number of animals	Nt748	Nt414	Nt267	Q204X	Combination code
209	0	0	0	0	0000 (CC0)
145	1	0	1	0	1010 (CC1)
168	1	1	0	0	1100 (CC2)
139	1	1	0	1	1101 (CC3)
43	2	0	1	0	2010 (CC4)
30	2	0	2	0	2020 (CC5)
59	2	1	0	0	2100 (CC6)
53	2	1	0	1	2101 (CC7)
60	2	1	1	0	2110 (CC8)
42	2	1	1	1	2111 (CC9)
29	2	2	0	0	2200 (CC10)
63	2	2	0	1	2201 (CC11)

^1^ 0: homozygous for normal alleles at the specific locus; 1: heterozygous carrier of the specific variant; 2: homozygous for affected alleles at the specific locus

The cumulative effects of the *MSTN* variants on the traits in the SA Bonsmara population were estimated using the General Linear Model (GLM) procedure in SPSS version 27. The model included the *MSTN* genotypes as a fixed effect, and the growth traits as dependent variables. Dunnett’s test in the GLM procedure was used to estimate mean differences as this test treats one group as a control and compare all other groups against it. The combination 0000 (animals that did not carry any of the four variants’ affected alleles) was allocated as the control. Significant differences were considered at *p* < 0.05.

## Results

### Genotypic frequencies

Thirteen *MSTN* variants were routinely included in the SNP array used for genotyping of the Bonsmara cattle in this study. For nine of the thirteen variants (Nt821, F94L, C313Y, D182N, E226X, Nt419, S105C, Nt324, and E291X), no affected alleles were observed. However, affected alleles were identified for Nt748, Nt414, Nt267 and Q204X. The calculated genotypic frequencies for the four affected variants were summarized in Table 1. The variant Nt748 was the most common in both heterozygous carrier (48.82%) and homozygous affected (35.02%) animals, while the Q204X variant occurred in only 1.18% of the population in a homozygous affected form (Table 2).

**Table 2:** Genotype frequencies of *MSTN* variants detected in the SA Bonsmara animals

Variant	Number of animals	Genotyped animals		
		Homozygous normal (%)	Heterozygous carrier (%)	Homozygous affected (%)
Nt748	1378	236 (17.13)	665 (48.26)	477 (34.61)
Nt414	1260	623 (49.44)	535 (42.46)	102 (8.10)
Nt267	1419	1043 (73.50)	344 (24.24)	32 (2.26)
Q204X	1778	1261 (70.92)	496 (27.89)	21 (1.18)

Bold values indicate > 40% heterozygous carriers and > 5% homozygous affected animals

### Association analysis

In Table 3, the GEBVs for the different growth traits are shown for clean, heterozygous and homozygous affected animals per variant. The Q204X variant significantly (*p* < 0.05) affected BW_DIR_ and WW_DIR_ causing an increase, and reduced FCR in heterozygous carrier animals. The traits WW_DIR_, ADG and FCR were significantly affected (*p* < 0.05) by both Nt748 and Nt414 variants. The Nt748 variant increased the WW_DIR_ and ADG, and decreased FCR in homozygous affected animals. Conversely, the Nt414 variant decreased the WW_DIR_ and ADG, and increased FCR of the homozygous affected animals. The Nt267 genetic variant had no significant effect (*p* > 0.05) on any of the growth traits analyzed.

**Table 3:** Mean ± Standard error (SE) GEBVs of growth traits for Bonsmara animals carrying affected alleles per *MSTN* variant

Genetic variant	Genotype	*N*	GEBVs for growth traits			
			BW_DIR_ (kg), Mean ± SE	WW_DIR_ (kg), Mean ± SE	ADG (kg), Mean ± SE	FCR (kg/kg), Mean ± SE
Nt748	0	236	1.25 ± 0.21	15.62^a^ ± 0.88	115.02^a^ ± 8.53	-44.07^a^ ± 2.99
	1	665	1.13 ± 0.15	15.13 ^a^± 0.63	109.57^a^ ± 6.11	-45.41^a^ ± 2.14
	2	477	1.35 ± 0.11	16.22^b^ ± 0.46	123.30^b^ ± 4.44	-51.74^b^ ± 1.56
Nt414	0	623	1.30 ± 0.11	16.09^a^ ± 0.49	124.26^a^ ± 4.72	-51.18^a^ ± 1.66
	1	535	1.28 ± 0.14	16.21^a^ ± 0.61	120.75^a^ ± 5.90	-50.04^a^ ± 2.07
	2	102	1.14 ± 0.23	14.67^b^ ± 0.99	102.87^b^ ± 9.64	-39.99^b^ ± 3.39
Nt267	0	1043	1.36 ± 0.07	16.23 ± 0.28	124.30 ± 2.72	-50.45 ± 0.95
	1	344	1.34 ± 0.13	16.13 ± 0.54	119.21 ± 5.32	-48.80 ± 1.87
	2	32	1.03 ± 0.31	14.61 ± 1.32	104.37 ± 12.80	-41.96 ± 4.49
Q204X	0	1261	1.00^a^ ± 0.14	14.95^a^ ± 0.60	114.22 ± 5.84	-43.74^a^ ± 2.05
	1	496	1.48^b^ ± 0.15	16.37^b^ ± 0.66	117.70 ± 6.37	-50.39^b^ ± 2.24
	2	0	0.00 ± 0.00	0.00 ± 0.00	0.00 ± 0.00	0.00 ± 0.00

^a, b^ means in the same column bearing the different superscript differ significantly at *p* < 0.05 per variant

0: Homozygous normal, 1: Heterozygous carrier, 2: Homozygous affected

N: Number of animals genotyped,

SE: Standard Error, Kg: kilograms,

BW: birth weight, WW: weaning weight, ADG: average daily gain, FCR: feed conversion ratio

### Additive effect of MSTN variants on growth traits in the SA Bonsmara

Table 4 illustrates the results for the growth traits assessed for the combined genetic variants in the Bonsmara population. The GEBVs of animals with the specific *MSTN* variant combinations were compared to those of animals carrying no affected mutations (CC0, “clean animals”). None of the 11 combined genotypes showed a significant effect on ADG (*p* > 0.05). Combination CC7 was associated with an increase in both BW_DIR_ and WW_DIR_, and a reduced FCR (*p* < 0.05). Combinations CC3, CC6 and CC9 were also favorable associated (*p* < 0.05) with a decreased FCR.

**Table 4:** Mean ± Standard error (SE) of GEBVs for growth traits assessed in relation to the combination of four genetic variants in the Bonsmara *MSTN* gene

Genotype combinations	*N*		GEBVs for growth traits			
		BW_DIR_ (kg), Mean ± SE	WW_DIR_ (kg), Mean ± SE	ADG (kg), Mean ± SE	FCR (kg/kg), Mean ± SE	
CC0	2096	1.18^a^ ± 0.09	15.86^a^ ± 0.42	129.17 ± 4.11	-47.57^a^ ± 1.43	
CC1	145	1.08^a^ ± 0.12	15.63^a^ ± 0.50	122.68 ± 4.93	-47.30^a^ ± 1.71	
CC2	168	1.02^a^ ± 0.11	15.35^a^ ± 0.47	118.44 ± 4.58	-47.41^a^ ± 1.59	
CC3	139	1.53^a^ ± 0.12	17.54^a^ ± 0.51	131.11 ± 5.04	-57.25^b^ ± 1.75	
CC4	43	1.38^a^ ± 0.21	17.24^a^ ± 0.93	144.32 ± 9.06	-56.91^a^ ± 3.15	
CC5	30	0.95^a^ ± 0.25	14.90^a^ ± 1.11	118.26 ± 10.84	-47.41^a^ ± 3.77	
CC6	59	1.42^a^ ± 0.18	17.05^a^ ± 0.79	139.93 ± 7.73	-56.36^b^ ± 2.69	
CC7	53	1.88^b^ ± 0.19	18.59^b^ ± 0.83	145.47 ± 8.16	-60.91^b^ ± 2.83	
CC8	60	0.95^a^ ± 0.18	15.83^a^ ± 0.78	125.31 ± 7.67	-51.50^a^ ± 2.66	
CC9	42	1.83^a^ ± 0.21	16.65^a^ ± 0.94	110.94 ± 9.16	-57.40^b^ ± 3.18	
CC10	29	1.55^a^ ± 0.26	16.63^a^ ± 1.13	125.92 ± 11.03	-49.12^a^ ± 3.83	
CC11	63	1.39^a^ ± 0.18	15.70^a^ ± 0.76	114.79 ± 7.48	-48.83^a^ ± 2.60	

The combinations are in the following order: Nt748, Nt414, Nt267 and Q204X

^a, b^ means in the same column bearing the different superscript differ significantly at *p* < 0.05 per variant

Bold indicates the group of animals that are significantly different from the control (CC0)

Kg: kilogram, SE: standard error

BW: birth weight, WW: weaning weight, ADG: average daily gain, FCR: feed conversion ratio

## Discussion

The ultimate goal of most modern beef cattle production systems is to increase production efficiency to remain competitive, economically viable, and meet the growing demand for meat. Both genomic data and reliable phenotypic recording of performance traits are required for genetic improvement of livestock species (Visser et al. 2020). The available performance recording systems, genomic tools, and advanced genetic evaluation methodology used to determine EBVs and GEBVs contributed to the genetic improvement of economically important traits in the beef cattle industry.

Different genetic variants that are significantly associated with growth traits in double muscled beef cattle have previously been identified in the *MSTN* gene. Previously, published literature reported the effect of various genetic variants including the most common variants such as Nt821, F94L and Q204X on growth traits such as birth weight, weaning weight, average daily gain, yearling weight, feed conversion ratio and various other growth traits in number of beef cattle breeds (Casas et al. 2004; Wang et al. 2015; Csürhés et al. 2023).

The current study revealed that the direct birth weight (BW_DIR_) of Bonsmara cattle was only affected by the Q204X variant, which resulted in increased BW_DIR_ in heterozygous carrier animals. Increased birth weight was also observed in heterozygous carriers of the Q204X variant (Allais et al. 2010; Csürhés et al. 2023 ) and Nt821 variant (Casas et al. 2004) in Charolais animals. These results follow the trend that has been previously reported by a number of studies (Hanset 1991; Arthur 1995; Bellinge et al. 2005; Wang et al. 2015). High direct birth weight has an impact on calf survival and is correlated with increased in incidence of dystocia, resulting in increased culling and decreased fertility (Bennett and Gregory 2001; Hickson et al. 2006).

In this study, WW_DIR_ of the Bonsmara cattle was significantly affected by Nt748, Nt414 and Q204X variants. The Q204X-heterozygous carriers in SA Bonsmara cattle had an average GEBV of 1.67 kg higher at weaning compared to the homozygous normal cattle. These results were consistent with findings of Csürhés et al. (2023), who reported that heterozygous carriers of the Q204X variant were 8.56 kg heavier at weaning than their counterparts in Charolais cattle. Similar increases in direct weaning weight of heterozygous carriers have previously been reported, for the C313Y variant in Piedmontese cattle (Casas et al. 1999) and the Nt821 variant in Charolais and Belgian Blue × British crossbred cattle (Casas et al. 2004). Mature cows that are heterozygous carriers of *MSTN* variants has the potential to produce heavier weaning weights while avoiding calving difficulties associated with double muscling (Casas et al. 1999; Csürhés et al. 2023).

Homozygous Bonsmara calves for the Nt748 variant in this study, had higher weaning weight (16.22 kg) GEBVs compared to their normal or heterozygous carrier counterparts. Similar observations were reported in Belgium Blue cattle (Arthur 1995) and Asturiana de los Valles (Cafion et al. 2002) cattle, where animals that were homozygous affected for the Nt821 mutation were also heavier at weaning, compared to homozygous normal animals. This study found that Nt414-homozygous affected Bonsmara calves had an average GEBV of 14.67 kg lighter at weaning, compared to homozygous normal cattle. However, no significant difference between the weaning weight of homozygous affected and homozygous normal animals, were reported for C313Y in Piedmontese (Casas et al. 1999), Nt821 in Belgian Blue × British crossbred animals (Casas et al. 2004) and F94L in Charolais (Csürhés et al. 2023).

Average daily gain (ADG) in the beef cattle industry is considered as a trait that influences production efficiency and profitability (Zhanga et al. 2016; Xu et al. 2019). The Nt748 variant increased ADG while Nt414 decreased ADG in homozygous affected Bonsmara animals compared to the homozygous normal individuals. The effect of the Nt748 variant in this study is similar to the effect of the F94L variant in Limousin cattle (Hales et al. 2020) and the Nt821 variant in Asturiana cattle (Cafion et al. 2002). The decreased ADG observed in this study in homozygous affected animals for the Nt414 variant was consistent with that reported for the Nt821 variant in Belgian Blue cattle (Meyermans et al. 2022). This supports previous suggestions that variants vary in effects among different breeds.

Feed Conversion Ratio (FCR) was significantly affected by the Nt748, Nt414 and Q204X variants. In both Nt748-homozygous affected and Q204X-heterozygous carrier animals in this study, FCR was favorably decreased, which was similar to the effect of the Nt821 variant in Belgian Blue cattle (Cundiff et al. 1998; Grobet et al. 1998; De Smet 2004; Boukha et al. 2011). Contrary to this, the Nt414 variant showed an unfavorable effect on FCR with increased FCR values in homozygous affected animals. Currently, there is no published research that is consistent with the results in this study. The reduced FCR in double-muscled animals are most likely owing to a shift in the composition of body weight gains toward more protein and less fat deposition, rather than changes in feed digestibility or maintenance requirements (De Smet 2004).

The impact of the combined *MSTN* variants on growth traits identified eleven combinations in which various numbers of affected *MSTN* alleles were observed in individual SA Bonsmara cattle. No comparable values are available in literature, but it is important to note that the genotypic combinations of several variants showed either favorable or unfavorable effects on the growth traits in this study. Genetic combination CC7, in which animals that are homozygous affected for the Nt748 variant and homozygous carriers for both the Nt414 and Q204X variants, was identified as the most superior combination resulting in increased weaning weight and decreased FCR. However, this combination will also cause an increase in birth weight, and should thus be approached with caution.

In this study, it was found that the Nt748-homozygous affected and Q204X-heterozygous carrier animals had improved WW_DIR_, ADG and FCR while Nt414-homozygous affected animals were inferior for these same traits. The Nt748 variant was identified as the only variant in the Bonsmara population to increase weaning weight without causing an associated increase in birth weight. The confounding effects between *MSTN* variants and breed are the most likely cause of these contradictory results.

## Conclusion

The results of this study improve the current understanding of the variability of the *MSTN* gene variants. It was shown that differing variants have either a favorable or unfavorable effect on growth traits in the SA Bonsmara breed. In addition, the combined genotypes revealed that there is an additive effect on economically important traits when more than one variant are present in the *MSTN* gene. These results will assist breeders to select for or against specific *MSTN* variants or combinations of variants in their breeding programs to improve production. It is recommended that farmers should perform DNA-based diagnostic testing for their animals and identify the specific variants that occur within their herds, before selection and mating decisions could be made.

## Data Availability

Data is available upon request.
